# High Level of Knowledge about Tungiasis but Little Translation into Control Practices in Karamoja, Northeastern Uganda

**DOI:** 10.3390/tropicalmed8090425

**Published:** 2023-08-24

**Authors:** Marlene Thielecke, Hannah McNeilly, Francis Mutebi, Mike B. Banalyaki, Rebecca Arono, Susanne Wiese, Felix Reichert, George Mukone, Hermann Feldmeier

**Affiliations:** 1 Charité Center for Global Health, Institute of International Health, Charité University Medicine, 13353 Berlin, Germany; 2 Edinburgh Medical School: Biomedical Sciences, The University of Edinburgh, Edinburgh EH8 9AG, UK; 3 Innovations for Tropical Disease Elimination (IFOTRODE), Kampala P.O. Box 24461, Uganda; 4 School of Veterinary Medicine and Animal Resources, College of Veterinary Medicine, Animal Resources and Biosecurity, Makerere University, Kampala P.O. Box 7062, Uganda; 5 Institute of Microbiology, Infectious Diseases and Immunology, Charité University Medicine, 12203 Berlin, Germany; 6 Department of Infectious Disease Epidemiology, Robert Koch Institute, 13353 Berlin, Germany

**Keywords:** tungiasis, neglected tropical disease, knowledge, attitude practices, health promotion, Uganda

## Abstract

Tungiasis is a neglected tropical disease (NTD) that can cause significant suffering and disability. Health promotion is an important pillar in NTD control programs, assuming that better knowledge contributes to reduced risk behavior and reduced risk of infection. The study objective was to assess tungiasis-related knowledge and its translation into control practices in a rural and highly endemic setting in Karamoja, Northeastern Uganda. We applied a mixed-methods design on household and community level. A semi-quantitative questionnaire on knowledge, practices, and attitudes (KAP) regarding tungiasis was administered to 1329 individuals with the main caring responsibilities in the household. Additionally, eight community dialogue meetings were held and analyzed. Overall, knowledge of tungiasis in humans was high but knowledge of tungiasis in animals was low. Most questionnaire respondents knew the causative agent and clinical presentations of tungiasis in humans, risk factors, and preventive measures. This tungiasis-related knowledge was translated into simple prevention measures. However, adequate tungiasis control was impeded due to a lack of resources, such as access to water and effective medical treatment. In conclusion, health promotion campaigns should be integrated with support towards adequate tungiasis control measures, such as provision of safe treatment, hardening of non-solid floors in the houses, and improved access to water.

## 1. Introduction

Tungiasis (sand flea disease) is a tropical skin parasitosis, caused by the penetration of the female sand flea *Tunga penetrans,* called jigger flea in East Africa, into the epidermis of its host. It is a zoonosis that affects humans and various domestic and sylvatic animals [1,2,3,4,5]. Frequent symptoms are pain and itching, and bacterial superinfections are common [6,7,8,9]. Repeated infections and/or mechanical manipulation of lesions can lead to the deformation of toes and eventually to disability [6,8,10,11]. The incidence of tungiasis usually increases during the dry season [12]. The off-host life cycle (involving eggs, larvae, pupae, and adult fleas) typically occurs in loose, dry, shaded, and organic-material-containing soil [13,14]. Off-host stages have been identified outdoors, particularly at the resting places of domestic animals, as well as inside houses with poor construction characteristics and non-solid floors [14]. Risk-factor studies in Brazil, Nigeria, and Kenya identified living in houses with earthen floors and mud walls as independent risk factors for the presence of tungiasis [15,16,17]. The smoothening of floors and regular sweeping of the house and compound are therefore considered preventive measures [18]. Furthermore, limited access to water is a risk factor, and daily washing of feet with soap has been shown to be protective against tungiasis infection [15,16,17].

In 2014, an effective, safe, and pain-free treatment for tungiasis was identified: the topical application of a formula of dimeticone oils with a low viscosity (NYDA^®^) [19]. NYDA^®^ seals the entrance to vital organs located in the so-called abdominal rear cone of the flea, namely the respiratory, intestinal, and genital tract [20]. So far, this mixture of dimeticone oils is not available in most endemic settings. Therefore, in an act of despair, people try to remove embedded sand fleas with sharp, non-sterile instruments, such as thorns, needles, and safety pins [18,21]. Mechanical extraction is not only painful and leaves deep wounds but is associated with a high risk of secondary bacterial infections, as well as transmitting blood-borne pathogens like hepatitis B and C viruses when instruments are shared [7,8,22,23]. In Uganda, the Ministry of Health recommends the topical use of benzyl benzoate emulsion (BBE) 25% *w*/*v*, a drug used for the treatment of scabies, although the efficacy and safety of BBE treatment for tungiasis has never been assessed. Regarding the treatment of tungiasis in animals, there are no recommendations at the national or international level so far.

Tungiasis is one of the most neglected and stigmatized NTDs, since it is strongly associated with poverty, highly visible, and leads to a characteristic gait after years of infection [24,25]. Affected communities frequently have little or no access to medical care and formal education [15,16,26]. One of the foundational pillars in the WHO road map for NTD control 2021–2030 is the inclusion of health programs such as WASH and health promotion into control programs [27,28]. Health promotion is an important, low-cost and relatively easy-to-implement part of control programs for NTDs, assuming that better knowledge about transmission can influence positive behavioral change, which in the long run reduces the risks of infection. For example, several studies on lymphatic filariasis showed that a high level of knowledge was associated with better compliance and more effective implementation of control measures [29,30,31]. Regarding tungiasis control, two different analyses using mathematical models established that public health promotion is an effective control measure [32,33].

The objectives of this study were to explore knowledge and opinions about tungiasis in the Karamojong people living in a remote rural and highly endemic area of Northeastern Uganda [26] and to identify barriers to tungiasis control in the study area.

## 2. Materials and Methods

### 2.1. Study Area and Study Population

The study was conducted in Napak district, Karamoja region, Northeastern Uganda, prior to the implementation of a project attempting to control tungiasis based on a novel treatment rationale combined with a One Health strategy. Napak district has seven sub-counties, which are further divided into 28 parishes and 138 villages. Based on information from the District Health Office, we selected the three adjacent parishes, Nagule-Angolol, Naitakwae and Nawaikorot, located in Ngoleriet sub-county, in which the prevalence of tungiasis was assumed to be around 50%. All 17 villages in the three parishes were included. A total population of 5482 individuals were registered during the project’s baseline assessment in February/March 2021 [26]. In the study area, the prevalence of tungiasis in the general population was 62.8% at the time of data collection [26].

The Karamojong people live as semi-nomadic pastoralists under extremely poor conditions [34]. They live in round huts which are less than 10 m^2^ and of low height. The roofs are made of straw, the walls of wooden sticks, and the floor of compacted or loose earth. Bedsteads consist of mats, rags, or animal skins directly on the floor. People live in *manyatas*, clusters of households surrounded by thorny hedges or bundles of sticks as protection against wild animals and raiders. Throughout the year, men and male adolescents wander through the savannah with their cattle, goats, and sheep searching for water and feed for their animals. In the *manyatas*, childcare and household chores are mostly performed by women and female adolescents.

**Figure 1:**
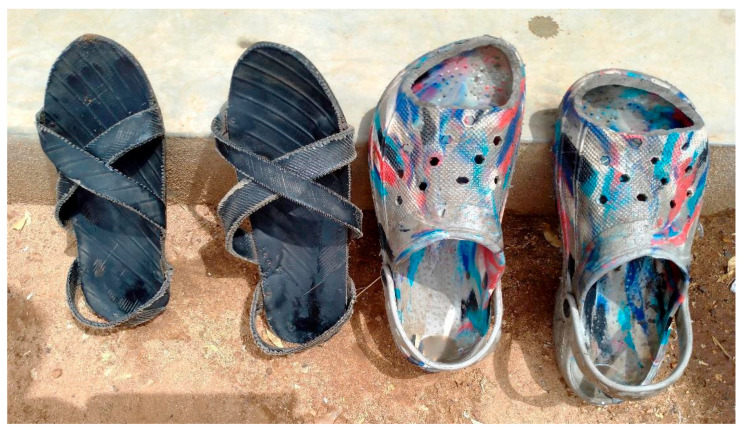
The left pair of sandals is made locally from tires, the right pair is made of plastic and originates from China.

Sorghum and sunflowers are planted on a small scale. The harvest is usually at the end of the dry seasons, of which there are two: one from December to March, the other from June to September. Some of the produce, as well as charcoal, is sold by women at small local markets [26]. Most of the adult Karamojong wear open sandals made of used car tires with thick and durable soles that protect their feet against thorny trees and shrubs which are common in the area. Men usually wear sandals of better quality than women, and very few children wear footwear (Francis Mutebi and Mike B. Banalyaki, unpublished observation 2022). In recent years, plastic sandals imported from China have been used (Figure 1). Water sources are shared community boreholes or taps, some of which are far away from villages and frequently broken. Accessible primary health care facilities are virtually non-existent.

### 2.2. Study Design

The study was based on a mixed-methods design, composed of a survey on knowledge, attitudes, and practices (KAP) on a household level, and eight public dialogue meetings on a community level.

The KAP study was conducted as a cross-sectional door-to-door survey in the 17 villages of the study area between February and September 2021. For each household, we included the main caretaker in the questionnaire survey. The main caretaker was defined as the person who oversees the welfare of all family members and who has the main caring responsibilities in terms of maintaining sanitation and hygiene. Usually these are adults, but in some households an older child had taken on responsibility, usually when the parents were too old or sick to take care of the families.

The semi-quantitative questionnaire included 50 questions covering the following categories:

- Knowledge: Questions on cause, risk factors for infection, symptoms and signs, prevention and treatment of tungiasis in humans and animals.
- Practices: Questions on activities regarding the treatment and prevention of tungiasis in humans and animals.
- Attitudes: Questions with a focus on stigma and the impairment of life quality.

The questionnaire included binary (yes/no option), multiple choice, and open questions. Five enumeration questions asked for a minimum number of possible answers (e.g., name at least four clinical complaints) (Supplementary File S1). The KAP questionnaire was pre-tested and improved in a pilot study in January 2021 conducted with 15 individuals from the same population in the parish of Nawaikorot. This article deals with questions related to tungiasis-related knowledge and corresponding control practices. Attitudes and tungiasis-associated stigma have been described elsewhere in a companion paper [25].

Socio-demographic data of all respondents were obtained. Following the questionnaire survey, a Village Tungiasis Health Worker (VTHW) examined the feet of each participant for the presence of tungiasis and assessed severity of the disease. VTHWs were local to the study area, literate, bilingual (NgaKarimojong and English), and had received 7 days of training from our team in conducting interviews and the diagnosis of tungiasis. Their work was supervised by two social workers and a project nurse and supported by existing state-established village health teams (VHTs) and Local Council One (LC1) leaders [26].

**Figure 2:**
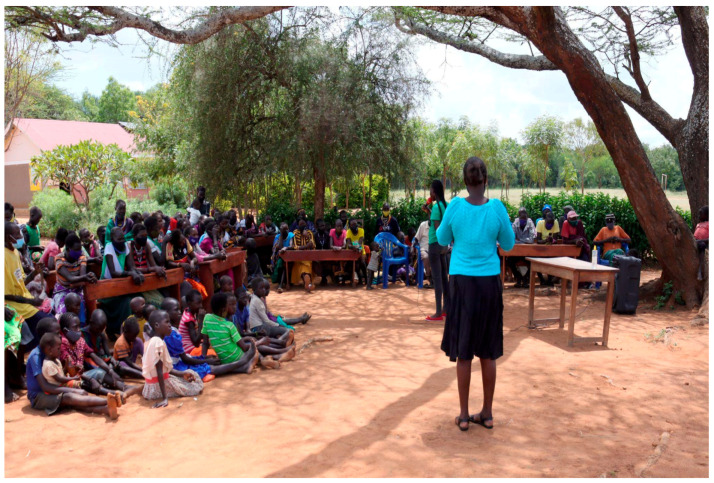
A community dialogue meeting is held in the presence of different stakeholders.

Eight dialogue meetings with the community about tungiasis and tungiasis control were held between 23 September 2021 and 1 October 2021 in central locations, such as schools or sub-county headquarters, that could readily be reached by the residents of the study area by foot (Figure 2). Mobilization for the meetings was carried out by the LC1 leaders, VHTs, and VTHWs. The meetings were announced a week in advance and residents were reminded by loudspeaker announcements one hour before the meetings started. The attendees were residents, community leaders (LC1 chairman, LCI representative), the parish chief, and government representatives of the sub-county. VTHWs and the team of the NGO IFOTRODE (Innovations for Tropical Disease Elimination) acted as facilitators and were backed up by district Health Officials who were actively involved in the discussion. The structure of the meetings consisted of an introduction, community consultation, discussion about opinions and knowledge on tungiasis, information about our study, and health education.

### 2.3. Data Collection and Analysis

KAP data were collected in the local language, NgaKarimojong, by bilingual VTHWs and recorded in English using open-source software on a smartphone (Open Data Kit v. 2022.4.3 available on free download from Google store, Get ODK Inc., San Diego, CA, USA). Questions were asked without reading out the predefined answer options, so that respondents could freely express their knowledge and understanding. The data collector then chose the option from the questionnaire that best fitted the given answer or summarized the response in an open-text field. The community dialogue meetings were also held in NgaKarimojong and recorded in the form of written minutes (using key quotes) in English by the VTHWs.

The questionnaire data were uploaded to a web-based database (ODK aggregate server) and then transferred to Microsoft Excel (2016) and RStudio (Version 1.2.5033 © 2009–2019, RStudio, Inc., Boston, MA, USA). The data were double checked for consistency prior to descriptive analysis. The evaluation of the five enumeration questions (quantitative data) was based on a previously defined list of possible correct answers. The enumeration questions also had the answer option “others” followed by free text. These answers were then individually scored as correct or incorrect and included in the overall count. The data evaluation of qualitative data (open-text responses and community dialogue meetings) was undertaken according to the qualitative content analysis described by Philipp Mayring and Thomas Fenzl [35]. Central to this method is the thematic clustering and categorization of individual statements to define the most relevant themes. Answers within the thematic categories were then counted. For the age of participants and the number of embedded sand fleas, the median was used as an indicator for the central tendency, and the dispersion of data was presented as interquartile ranges.

### 2.4. Ethical Considerations

Ethical approval for this study was provided by the Vector Control Division of the Ugandan Ministry of Health Ethical Committee (VCDREC112/UG-REC-018) and the Uganda National Council of Science and Technology (HS2623, July 2019). The District Chief Administrative Officer approved the study being carried out (CR/205/1). Before applying the questionnaire to study participants, the VTHWs explained the aim and methods of the study in simple words in the local language. Individuals had the opportunity to ask questions and make comments. Informed written consent was obtained from each study participant in NgaKarimojong language. Those who were unable to sign provided their fingerprint. In the case of children under 18 years of age, both the main adult caretaker and the minor were asked for written consent. Any individual could withdraw from the study at any time without providing reasons. Confidentiality was kept for all stored data from individuals.

## 3. Results

### 3.1. Socio-Demographic Data and Tungiasis Status

At the time of our data collection, 1338 households were registered in the study area. For each household, the individual with the main caring responsibilities was eligible to participate in the study. In total, 1329 individuals responded to the KAP questionnaire; nine did not consent. The demographic and socio-economic characteristics of the respondents have been published previously [25]. The vast majority (89.4%) were female. The median age was 44 years (min 9/max 115 years) (Nine respondents reported they were over 90 years old, which in this context may simply mean ‘very old’, as not everyone might know their exact age). Casual labor was the most frequent occupation (n = 574, 43.2%). The level of formal education among the respondents was very low, as 84.7% had never attended school. A total of 657 of our 1329 respondents (49.4%) stated to possess animals (Table 1).

A total of 811 of the 1329 respondents (61.0%) were diagnosed with tungiasis. They had a median number of 14 lesions (min 1/max 591; IQR 23), with live, dead, and manipulated sand fleas included. Most respondents with tungiasis had moderate infections with 6–30 sand flea lesions (n = 429, 52.9%), followed by mild infections with 1–5 lesions (n = 198, 24.4%), and heavy infections with above 30 lesions (n = 184, 22.7%).

### 3.2. Knowledge about Cause of Tungiasis and Disease Manifestation

In total, 1100 respondents (82.8%) knew that sand fleas are the causative agent of tungiasis. Local designations for jiggers are *ngikur* (‘something hiding that is taken out’), *ngimujai* (‘sweet feeling when scratching’), *edudut* (‘single jigger’), and *ngidudui* (‘more than one jigger’). Open-text answers showed that many people had very good knowledge about the parasite’s life cycle and biological characteristics of sand fleas. Some described in detail the enlargement of embedded sand fleas, knew about blood sucking, and the production and release of eggs—for example: *“[They] are small fleas that penetrate and dwell in the human skin hence forming a jigger in it and later start production of eggs”*.

**Figure 3:**
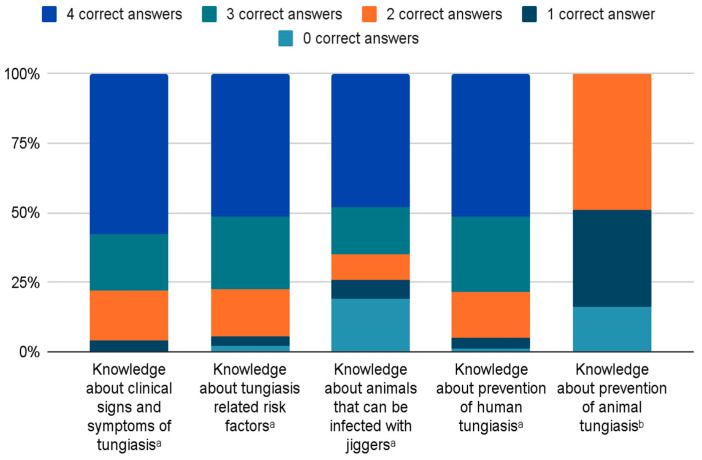
Number of correct answers in enumeration questions that ask for a minimum number of answers. ^a^ The number of correct answers ranged between 0 and 4, because multiple answers could be given and the respondent was asked to name four options if possible. ^b^ The number of correct answers ranged between 0 and 2, because multiple answers could be given and the respondent was asked to name two options if possible. Note: knowledge about the treatment of tungiasis in humans or respectively in animals is not depicted in this table, because it is difficult to determine what a correct answer is in this context (see Table 7 and Table 8).

When asked how people acquired tungiasis, 81.9% (n = 1089) responded “jiggers enter the skin”. A small number (n = 63, 5.1%) named witchcraft or cursing as a cause of jiggers. Almost all respondents (n = 1287, 96.8%) knew that tungiasis is not a blood-borne disease. More than half of all respondents (n = 766, 57.6%) could correctly name at least four clinical complaints (Figure 3). The most frequently mentioned were itching (96.8%), pain (83.9%), and swelling (54%) (Table 2). Only 0.4% could not name a single complaint (Figure 3).

### 3.3. Knowledge about Risk Factors and Animal Tungiasis

When asked how tungiasis was acquired, 51.2% (n = 680) of respondents could name at least four correct factors, and only 2.2% (n = 29) did not know a single one (Figure 3). Dirty floors (86.5%), poor body hygiene (71%), and poor housing (59.7%) were the most frequently given answers (Table 3). Lack of footwear was rarely mentioned (7.5%). A total of 90.6% of respondents (n = 1204) knew that the frequency of tungiasis increases with dry weather conditions. Seventy respondents associated high frequency of tungiasis with windy weather, harvesting, and grain husks on the ground, which are characteristic of the dry season; for example: *“[Jiggers are most common] during harvesting season due to the remains of sunflowers*”.

Regarding tungiasis in animals, 48.0% (n = 638) of respondents could name at least four animal species which may get tungiasis (Figure 3). Pigs and dogs were named most often (48.8% each) (Table 4). Notably, 19.0% (n = 252) could not name a single animal (Figure 3), half of whom owned animals (n = 129).

### 3.4. Knowledge and Practices concerning the Prevention of Human Tungiasis

When asked about prevention measures for humans, 51.6% (n = 686) of respondents could list at least four prevention measures, and only 1.3% (n = 17) could not name a single one (Figure 3). The most frequent answers were regular washing of the feet (90.4%) and keeping houses and compounds clean (76.2%) (Table 5). Wearing closed shoes was mentioned only by 10.8% as an effective preventive measure.

It was found that only 115 out of 1329 respondents (8.7%) reported possessing closed shoes and only five individuals wore them daily. The great majority of respondents reported daily sweeping of the floor in the house (n = 1110, 83.5%) and the compound (n = 1011, 76.1%). Regular washing of feet was also frequently stated: 93.8% (n = 1246) of the people said they washed their feet at least once a day. A total of 19.6% (n = 260) of the respondents confirmed using indoor spraying of insecticides as a preventive measure; 89.6% (n = 233) of those reported using the insecticide Dudu Dust^®^ (containing carbaryl). Only 13.5% (n = 180) of respondents said they allowed animals indoors, with cats named most often (n = 121, 67.2%).

### 3.5. Knowledge and Practices concerning the Prevention of Animal Tungiasis

Almost half of the respondents (n = 648, 48.8%) could correctly name at least two tungiasis-prevention measures for animals, while 16.3% (n = 217) could not name any (Figure 3). Keeping animal dwellings clean (54.5%) and spraying animals with insecticides (67%) were the most frequently named preventive measures (Table 6).

In the practice section of the KAP questionnaire, among animal owners (n = 657), the answers on the frequency of cleaning animal dwellings differed: 35.2% (n = 231) stated that their dwellings were cleaned at least every two days, while 45.4% (n = 298) reported cleaning their dwellings only rarely (n = 185, 28.2%) or never (n = 113, 17.2%).

**Table 6:** Knowledge about prevention of animal tungiasis (N = 1329).

Answer Options (Multiple Answers Possible)	Frequency of Answers	(%)
Spraying animals with insecticides	890	(67.0)
Keeping animal dwellings clean	724	(54.5)
I don’t know	214	(16.1)
Cementing the floor of animal dwellings	171	(12.9)
Others *	28	(2.1)

Enumeration question 5: Name at least two methods for jigger control/prevention in animals. * Others included applying herbal medicines, applying Dudu Dust^®^, greasing, smearing the house with cow dung, washing animals with OMO^®^ (a brand name of a common detergent in Uganda), washing animals with fermented animal urine, and giving dewormer.

### 3.6. Knowledge and Practices concerning the Treatment of Human Tungiasis

When asked about appropriate treatment methods for tungiasis in humans, 416 respondents (31.3%) named extraction as an appropriate means of treatment. Topical application of dimeticone was named by 13.5% of respondents (Table 7). The most frequent answers in the category “other answer options” were topical application of grease or oil (n = 32), application of ash (n = 19) and application of tobacco (n = 7).

**Figure 4:**
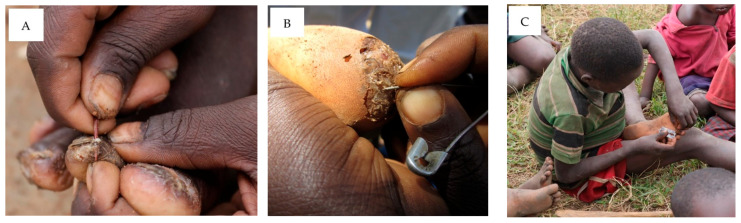
Instruments used for mechanical extraction of embedded sand fleas are, for example, thorns (**A**), safety pins (**B**), and razor blades (**C**).

In the practice section of the KAP questionnaire, it was shown that 89.8% (n = 1193) of the respondents used mechanical extraction of embedded sand fleas as a treatment method (Figure 4). (All further details about extraction of embedded sand fleas were collected from only 1119 respondents). Instruments used for extraction were razor blades, thorns, sharp pins, safety pins, and needles. Overall, 65.7% (n = 735) of respondents stated they neither boiled the instrument nor applied an antiseptic before extraction. After extraction, 14.3% (n = 160) of people reported applying antiseptics (not further specified) to the wounds. Moreover, many respondents described applying hot or cold ash (n = 342, 30.6%), tobacco (n = 296, 26.5%), or a mixture of both (sometimes mixed with other substances) to the wound after extraction. The application of greasy substances, like petroleum jelly, was also mentioned in the treatment of tungiasis: 67 (6.0%) of the people using the extraction of embedded sand fleas as treatment said they applied greasy substances afterwards. Application of greasy substances was also reported as a means to kill jiggers (14.8%, n = 197).

**Table 7:** Knowledge about treatment of human tungiasis (N = 1329).

Answer Options (Multiple Answers Possible)	Frequency of Answers	(%)
Mechanical extraction of embedded sand fleas	416	(31.3)
Cleaning wounds	251	(18.9)
Visiting health facility	235	(17.7)
Topical application of dimeticone	179	(13.5)
Others *	179	(13.5)
Topical application of BBE	126	(9.5)
I don’t know	114	(8.6)

Question: What is the appropriate method to treat jiggers in humans? (multiple answers possible). Note: Extraction of embedded sand fleas is only appropriate when working under sterile conditions. * Others included bathing/body hygiene (n = 78), applying greasy substances/oil (n = 32), applying ash (n = 19), applying tobacco (n = 7), applying Dudu Dust^®^, applying oil/castor oil, spraying with veterinary drugs, applying Sodom apple (Calotropis procera) juice.

### 3.7. Knowledge and Practices concerning the Treatment of Animal Tungiasis

When asked about treatment methods for tungiasis in animals, the topical application of unspecified veterinary insecticides was named by 65.8% (n = 875) and regular spraying of animals by 8.7% (n = 116) of respondents (Table 8). 22.3% (n = 297) did not know any tungiasis treatment for animals.

In the practice section of the KAP questionnaire, 30.3% (n = 403) of the respondents stated that they treated their animals when infected with jiggers. Of these, the majority (n = 239, 59.3%) said they applied agricultural chemicals (not further specified). Other relevant answers were the extraction of embedded sand fleas with sharp instruments (28.1%), and the application of other substances, e.g., grease (24.7%).

**Table 8:** Knowledge about treatment of animal tungiasis (N = 1329).

Answer Options (Multiple Answers Possible)	Frequency of Answers	(%)
Application of veterinary insecticide	875	(65.8)
I don’t know	297	(22.3)
Spraying of chemicals *	116	(8.7)
Topical application of dimeticone	98	(7.4)
Others **	69	(5.2)
Mechanical extraction of embedded sand fleas	19	(1.4)

Question: What is the appropriate method to treat jiggers in animals? (multiple answers possible). Note: Extraction of embedded sand fleas is only appropriate when working under sterile conditions. * Such as pesticides, like insecticides, sulfuric acid, Ambush^®^ (a brand name of a common crop pesticide in Uganda, containing 500 g/L permethrin), or OMO^®^. ** Others included applying greasy substances, applying ash, applying acid, and burning them with hot metal.

### 3.8. Community Dialogue Meetings

A total of 803 adults (162 men, 641 women) attended the eight meetings (mean 100, min 42, max 159). More women than men attended each of the meetings, with a mean ratio of 4:1 (ranging from 1:1 to 8:1). The number of attending children was not documented.

When the meeting participants were asked about their general knowledge of tungiasis, some could provide correct explanations of the causative agent and related clinical symptoms, e.g., *“A [jigger] flea is a very small living organism that lives in dust, enters the body and starts itching. When removed, it creates a wound”.* Some explained that sand fleas bred in dust and leftover husks from sorghum, sunflower, and maize cobs, e.g.,: *“Plenty of food caused jiggers some time ago, and sunflower”*, *“Keeping sunflower within the house [...] causes jiggers”*, and *“Dust causes jiggers”*. Also, some animals (mainly rats) were named as reservoirs for jiggers, e.g., *“rats bring jiggers”* and *“rats shed fleas in dusty places”*.

Furthermore, poor body hygiene, dirty houses, and old age were described as relevant factors in the occurrence of tungiasis: *“[Tungiasis is related to the] failure to bathe sometimes related to age, mostly old people [are affected]”*, *“Dirtiness causes jiggers”*, *“Jiggers come from dusty houses”*, and *“Elderly women suffer from jiggers as no one helps them to extract”*.

When the community were asked where they saw the problems and challenges in controlling tungiasis, residents pointed out that a lack of various resources was a barrier to achieving good hygiene and sanitation. They reported a lack of water for bathing and smearing houses; a lack of cow dung for smearing floors, as animals are always far from homes; and a lack of soap. One resident explained: *“[We] lack water for bathing and smearing houses as boreholes are broken and encroached by enemies”*. Some argued that sand fleas existed even if homes were smeared with cow dung or swept, as they hid in the walls.

At the end of the meetings, residents made several requests. They asked for soap, BBE, Dudu Dust^®^, products to kill mice and rats, beds, and some said that *“safety pins should be provided”* for the manual extraction of sand fleas. Some participants also requested that the community should be educated about risk factors of tungiasis and prevention methods, arguing that jiggers could be reduced through better knowledge about proper housing, body hygiene, and animal keeping. They suggested that heavily affected families should be identified and specifically educated.

## 4. Discussion

### 4.1. Knowledge about Human and Animal Tungiasis

Overall, knowledge about human tungiasis among the study population was high. The majority of respondents gave correct answers regarding the causative agent and clinical presentation of tungiasis, factors presumably associated with tungiasis, and appropriate preventive means. For the five enumeration questions that required a minimum number of answers, most respondents were able to comply. Our result of good tungiasis-related knowledge among the affected community is consistent with previous KAP studies on tungiasis in resource poor communities in Brazil, Kenya, and Uganda [21,36,37]. However, this finding contrasts with KAP studies on other skin NTDs, such as scabies, leprosy, lymphatic filariasis, and cutaneous leishmaniasis, where knowledge was mostly described as poor [24,38,39,40,41,42,43,44,45,46]. Unlike most other skin NTDs, in tungiasis the causative agent can be seen with the naked eye and it can be found obvious that penetrating sand fleas lead to sand flea disease. In addition, the extremely high prevalence of tungiasis in our study area of 62.8% [26] makes it very likely that our respondents acquired knowledge about tungiasis through experiences of tungiasis infection in themselves or family members.

Compared to the high level of knowledge about human tungiasis, our study revealed limited knowledge about animal tungiasis, as previously described in a KAP study in Bugiri District, Eastern Uganda [37]. In our study population, the three questions with the poorest response rates were about animals that can be infected with jiggers and about appropriate prevention and treatment methods for animal tungiasis. Of the 1329 respondents, 19.5%, 16.1%, and 22.3%, respectively, answered “I don’t know” (Table 4, Table 6 and Table 8). In comparison, the answer option “I don’t know” ranged between only 8.6% and 0.4% in the questions on human tungiasis (Table 2, Table 3, Table 5 and Table 7). However, many knew the fact that tungiasis is a zoonosis; 41.6% of respondents named close contact to animals as a risk factor, and 40.0% of respondents named keeping animals away from houses as a preventive measure.

A plausible explanation for the limited knowledge of animal tungiasis is that in our study area animals are less often and less severely affected and thus play a less important role as a reservoir than in other endemic areas [26]. The population of Karamoja mainly keeps cattle, sheep, and goats. Based on data from our baseline survey [26], these animals are rarely infected and seem to play a minimal role in the transmission dynamics. The most relevant animal hosts for sand fleas in other parts of Uganda are pigs [3], and also in our study area 8 out of 10 examined pigs (80%) were found infected with tungiasis [26]. Although there are few pigs in Karamoja, many of our respondents (n = 649, 48%) still mentioned them as animals which can be infected with jiggers. Furthermore, data from soil analysis indicate that most transmission of *T. penetrans* takes place inside houses (Mutebi et al., manuscript in preparation) where animals are rarely present. The vast majority of respondents stated that they would not keep animals inside their houses, except cats to catch rats and mice. Tungiasis in cats is known from Northeast Brazil [2,4] but has only rarely been found in Uganda [3,26]. Rats and mice were named as a cause for getting tungiasis in our KAP survey and were frequently associated with the occurrence of tungiasis among humans in the community dialogue meetings. However, in Uganda, rats and mice have not yet been studied systematically to assess their role as reservoirs. It is possible that they are merely indicators of poverty and poor hygienic conditions and thus indirectly associated with tungiasis. Also, it has to be considered that many inhabitants may not be able to differentiate between *T. penetrans* and other flea species and therefore, cat, rat or chicken fleas, such as *Echidnophaga gallinacean*, can be mistaken for sand fleas (Francis Mutebi, unpublished observation).

The biggest knowledge gap we identified in our study population was related to adequate treatment of tungiasis. Lack of knowledge about safe treatment options such as dimeticone, is certainly due to the fact that it had not previously been available in the region. Accordingly, it is noteworthy that 13.5% of respondents indicated dimeticone as a treatment option. To the best of our knowledge, the communities had never been formally educated on tungiasis prior to conducting the KAP study. However, since our study took several months to be completed, households which were visited last could probably have gotten some information from those who were visited first and who had already been treated.

### 4.2. Shoes as a Controversial Preventive Measure

Risk-factor studies in Kenya and Nigeria show that the lack of shoes increases the risk of acquiring tungiasis [16,47]. A cross-sectional study with Kenyan children identified walking barefooted (OR = 3.28; 95% CI: 1.78–6.04) to be associated with tungiasis [48]. A meta-analysis showed that use of closed footwear was significantly associated with a lower odds of certain soil-transmitted skin NTDs, including tungiasis (OR = 0.42; 95% CI: 0.26–0.70) [49].

Therefore, it would be expected that wearing closed shoes as a protective barrier against penetrating sand fleas would also be an obvious measure of tungiasis control in the community in Napak District. But interestingly, only a few of our study participants named the lack of shoes as a risk factor and wearing shoes as a protective means. A previous tungiasis-prevention study in Madagascar showed that various practical, cultural, and medical factors discouraged people with tungiasis from regularly wearing shoes, even when they had been distributed to them earlier [50]. Study participants in Madagascar reported discomfort, perspiration, unpleasant odor, and even pain when wearing closed shoes, especially if they were heavily infected with sand fleas [50]. Other reasons were impracticability in daily activities given the residents’ high exposure to water and wet soil, fear of being easily recognized as someone having tungiasis, and reluctance to wear out the shoes which were of great material value to them [50].

A possible reason for why closed shoes were not seen as a relevant means of tungiasis prevention in our study in Uganda, could be that they are hard to access and very rare in this community. But even if closed shoes were made available, there are still practical challenges on site. First, the geographical terrain of the study area is very rough, consisting of stones, brushwood, and thorns. Second, the Karamojong have long walking distances to graze their animals, to market their products or to search for food. Only closed shoes that can withstand the stress of rough underground and intensive use are useful, regarding sustainability and affordability. The open shoes that the Karamojong usually use have the advantage that they are easy to put on and that heat does not accumulate. Closed shoes might be perceived as impractical in these regards. Another challenge regarding the implementation of closed shoes as a preventive means is the fact that footwear is removed inside the houses, because being barefoot is more comfortable, particularly while sleeping; people want to keep the house clean; and it is a widespread practice in Uganda (Francis Mutebi and Mike B. Banalyaki, unpublished observation). Given the assumption that the main transmission of tungiasis occurs indoors (Mutebi et al., manuscript in preparation), the unprotected feet are then exposed to penetrating sand fleas.

### 4.3. Translation of Knowledge into Practice

An example of the successful translation of knowledge into practice concerns the widely practiced preventive daily washing of feet and frequent cleaning of house and compound. Most respondents stated that they cleaned their house and compound regularly, as well as washing their feet daily [25].

An example of unsuccessful translation of knowledge into practice concerns the extraction of embedded sand fleas with sharp instruments. While 31.3% of respondents named extraction as an appropriate method, the practice assessment showed that almost three times as many (89.8%) used this method. At the time of our KAP study, dimeticone was not available in the study area, which explains why so many of the respondents had to resort to mechanical extraction of embedded sand fleas. Following the interventions related to our One Health approach and the supply of dimeticone, we expect better knowledge about its effectiveness and less need for painful and hazardous extraction of sand fleas.

Another control method that was frequently described was indoor spraying of insecticides as a preventive means for both humans and animals. The vast majority named Dudu Dust^®^, a broad-spectrum insecticide that contains carbaryl, a cholinesterase inhibitor which is highly toxic for humans and was classified as a likely human carcinogen by the United States Environmental Protection Agency [51]. Furthermore, application of greasy substances, such as petroleum jelly, was repeatedly mentioned in the practice assessment regarding treatment of human tungiasis. These substances have a very low melting point and rapidly turn into oil when applied on the skin, particularly in hot climates. Liquid fatty acids may thereby creep into the airways of the embedded sand flea and suffocate the parasite. We already suspected that the application of Vaseline could have a therapeutic effect in part in a previous study, when we compared the application of dimeticone with bathing in KMnO4 with subsequent application of petroleum jelly [19]. Also, two small case studies with two and eight patients have shown an effect of petroleum jelly application on embedded sand fleas [52,53].

Our study highlights that implementation of control practices requires not only a good level of knowledge, but also resources and the availability of alternatives, which is supported by KAP studies on tungiasis from Brazil and Kenya [21,36]. Despite good knowledge, individuals can be severely affected with tungiasis, as a case series in Colombia shows [54]. To consider which control measures are effective, accepted, and sustainable in a particular setting, the assessment of local knowledge and dialogue with the community needs to be a priority [55,56]. Glenn et al. (2020) describe this approach in the control of NTDs as bottom-up, instead of top-down, and pro-active, involving multiple stakeholders, above all the affected community [57].

In the community dialogue meetings, participating community members emphasized body hygiene and a clean home and environment for tungiasis control, and discussed lack of material (cow dung) for hardening house floors and lack of water as barriers. In addition to the provision of safe treatment, such as dimeticone, other potential interventions in this area should therefore focus on establishing (1) better floors in people’s houses and (2) better access to water.

Elson et al. (2017) suggest replacing loose floors with solid floors made of concrete, ceramics, or bricks as an appropriate measure to improve cleanability and minimize occurrence of sand fleas and their off-host stages [18], though it depends on the socio-cultural context if this is an adequate option. In our semi-nomadic rural study population, the application of cow dung on the floor, which is highly regarded in the community, may be a more appropriate, cheap, and sustainable method of sealing the ground. However, cow dung is rarely available in the villages because most of the time the animals are grazing in the savanna. Moreover, the effectiveness of smearing cow dung for tungiasis prevention has not been researched so far.

As water is essential for preventive tungiasis control, a sufficient number of intact wells or other water sources must be established. Likewise, better access to soap would facilitate tungiasis prevention [16,17]. In the community dialogue meeting, these points were also raised and the participants requested soap and water sources.

### 4.4. Study Limitations

It needs to be considered that self-reported information about prevention and treatment practice is based on statements and claims of the respondents and not on objective observations. In particular, the information on the application of hygiene measures (washing of feet and cleaning of house and compound) may have been overstated. Some of the data collectors assumed that due to the limited access to water many of the people washed their feet and cleaned their houses less often than stated (unpublished observation). Further, we acknowledge that there is a risk of misinterpretation of the given responses, as our local data collectors translated them from NgaKarimojong into English.

Another study limitation is that the usage of chemicals for tungiasis control in animals (referred nonspecifically in the questionnaire as veterinary insecticides or agrochemical substances) was not investigated in depth. Also, the practice assessment had few questions on the prevention and treatment of animal tungiasis. Here, a further and more targeted investigation and survey would be worthwhile.

## 5. Conclusions

The population of this highly endemic area demonstrated good knowledge about human tungiasis, but limited resources hindered the translation of knowledge into adequate control practices. Therefore, health promotion alone is not likely to result in effective tungiasis control in the presented context. The provision of effective treatment such as dimeticone, and the improvement of infrastructure, like optimization of floors in the houses and accessibility to water, should be addressed. Closed shoes were not considered a relevant preventive measure in the community. When implementing control measures, local knowledge and dialogue with the community are of key importance.

## Figures and Tables

**Table 1:** Possession of domestic animals (N = 1329).

Answer Options (Multiple Answers Possible)	Number	(%)
No animals	672	(50.6)
Chickens	372	(28.0)
Goats	357	(26.9)
Cattle	352	(26.5)
Sheep	289	(21.7)
Cats	152	(11.4)
Other poultry *	136	(10.2)
Dogs	124	(9.3)
Pigs	12	(0.9)
Other animals **	12	(0.9)

* Other poultry included ducks, pigeons and turkeys. ** Other animals, such as donkeys.

**Table 2:** Knowledge about signs and symptoms of tungiasis (N = 1329).

Answer Options (Multiple Answers Possible)	Frequency of Answers	(%)
Itching	1287	(96.8)
Pain	1115	(83.9)
Swelling	718	(54.0)
Loss of nails	350	(26.3)
Ulcers	325	(24.5)
Warm skin	272	(20.5)
Loss of toes	240	(18.1)
Deformed feet	98	(7.4)
Alteration of gait	93	(7.0)
Others *	37	(2.8)
Bacterial superinfection	33	(2.5)
I don’t know	5	(0.4)

Enumeration question 1: Name at least 4 complaints of having jiggers. * Others included appearance of fluid, anemia, sleeping difficulties, loss of appetite, weight loss, and wounds with bad smell.

**Table 3:** Knowledge about factors presumably associated with tungiasis (N = 1329).

Answer Options (Multiple Answers Possible)	Frequency of Answers	(%)
Dry/dusty/dirty floor	1150	(86.5)
Poor body hygiene	943	(71.0)
Poor housing	793	(59.7)
Close contact to animals	553	(41.6)
Dry weather	362	(27.2)
Sleeping on the floor	196	(14.7)
Overcrowded homes	125	(9.4)
No footwear	100	(7.5)
Waste on the compound	90	(6.8)
Others *	28	(2.1)
Presence of rats/mice	26	(2.0)
I don’t know	22	(1.7)
Open defecation	11	(0.8)

Enumeration question 2: Name at least 4 factors that make some people or families or homes to be more affected by jiggers than others. * Others included remains and husks of sunflowers or cereals, poverty, blood group, and God.

**Table 4:** Knowledge about animals that can be infected with jiggers (N = 1329).

Answer Options (Multiple Answers Possible)	Frequency of Answers	(%)
Pigs	649	(48.8)
Dogs	648	(48.8)
Goats	582	(43.8)
Cattle	519	(39.1)
Cats	501	(37.7)
Sheep	436	(32.8)
Chicken	307	(23.1)
I don’t know	259	(19.5)
Other animals *	21	(1.6)
Donkeys	13	(1.0)

Enumeration question 3: Name at least 4 animals which can be infected with jiggers. * Other animals included rats, mice, and ducks.

**Table 5:** Knowledge about prevention of human tungiasis (N = 1329).

Answer Options (Multiple Answers Possible)	Frequency of Answers	(%)
Regular washing of the feet	1202	(90.4)
Keeping houses/compound clean	1013	(76.2)
Apply concrete/cow dung on the floor	574	(43.2)
Keep animals away from houses	531	(40.0)
Proper waste disposal	475	(35.7)
Spraying the house with insecticides	447	(33.6)
Wearing shoes	143	(10.8)
Others *	25	(1.9)
I don’t know	15	(1.1)

Enumeration question 4: Name at least 4 methods for jigger control/prevention in humans. * Others included applying grease/oil/paraffin to the feet, killing rats in the house.

## Data Availability

The data presented in this study are available on request from the corresponding author.

## Supplementary Materials

The following supporting information can be downloaded at: https://www.mdpi.com/article/10.3390/tropicalmed8090425/s1, File S1. Selected Questions from KAP Questionnaire.
