# Supplementary material for: High Level of Knowledge about Tungiasis but Little Translation into Control Practices in Karamoja, Northeastern Uganda
Source: Trop Med Infect Dis. 2023 Aug 24;8(9):425. doi: 10.3390/tropicalmed8090425 (PMC10537667; doi:10.3390/tropicalmed8090425)
Supplement: Supplementary file 1 [file tropicalmed-08-00425-s001.zip › tropicalmed-2521844-supplementary.pdf]

SUPPLEMENT: Selected Questions from KAP Questionnaire

KNOWLEDGE

1. What causes/is tungiasis/(infestation with) jiggers? (Open text box)
2. How do people get jiggers? (Response options: 0=I don't know; 1=cursing; 2=witchcraft; 3=jiggers entering the skin; 88=other (Open text box))
3. Do you think jiggers can be passed on from the parents to their newborn children via blood? (Y/N)
4. Which season of the year do you think jiggers are most common? (Open text box)
5. Name at least 4 risk factors (factors which increase the chances) for getting jiggers. (Response options: 0=I don't know; 1=dry/dusty/dirty floor; 2=poor housing; 3=poor body hygiene; 4=living/working with animals; 5= no footwear; 6=dry weather conditions; 7=sleeping on the floor; 8=overcrowded homes; 9=open defecation; 10=disposal of waste on the compound; 88=other (Open text box))
6. Name at least 4 animals which can be infected with jiggers. (Response options: 0=I don't know; 1=Cattle; 2=Pigs; 3= dogs; 4=cats; 5=chickens; 6=goats; 7=sheep; 8=donkeys; 88=other (Open text box))
7. Name at least 4 symptoms/clinical signs of jigger infestation in humans. (Response options: 0=I don't know; 1=Itching; 2=pain; 3=swelling; 4=ulcers; 5=bacterial superinfection; 7=loss of nails; 8=loss of toes; 9=alteration of gait; 10=deformed feet; 88=other (Open text box))
8. Name at least 4 methods for jigger control/prevention in humans. (Response options: 0=I don't know; 1=regular washing of the feet; 2=proper waste disposal; 3=keeping houses/compound clean; 4=keep animals away from compounds/human houses; 5=spraying the house with insecticides; 6=wearing shoes; 7=apply concrete/cow dung on the floor; 8=eliminating dust from floors (sweeping); 88=other (Open text box))
9. State at least two methods for jigger control/prevention in animals. (Response options: 0=I don't know; 1=keep animal dwellings clean; 2=regular spraying of animals with insecticides; 3=cementing the floor of animal dwellings; 88=other (Open text box))
10. What is the appropriate method to treat jiggers in humans? (Response options: 0=I don't know; 1=topical application of dimeticone; 2=topical application of BBE on the embedded sand flea; 3=visit of health facility for seeking help; 4=cleaning wounds; 5=mechanical extraction of embedded sand fleas; 88=other (Open text box))

11. What is the appropriate method to treat jiggers in animals? (Response options: 0=I don't know; 1=topical application of dimeticone; 2=topical application of veterinary insecticide on the embedded sand flea; 3=mechanical extraction of embedded sand fleas; 88=other (Open text box))

PRACTICES

1. How often do you wash your feet? (Response options: 4=several times per day; 3=once per day; 2=every other day; 1=several times per week; 0=fewer times)
2. Do you possess shoes? (Y/N)

   If yes, How often do you wear the shoes? (Response options: 3=daily; 2=some days per week; 1=occasionally; 0=never)
3. How often do you sweep your house? (Response options: 4=every day; 3=every other day; 2=weekly; 1=fewer times; 0=never)
4. How often do you sweep the compound? (Response options: 4=every day; 3=every other day; 2=weekly; 1=fewer times; 0=never)
5. How often do you clean the dwellings of your animals? (Response options: 4=every day; 3=every other day; 2=weekly; 1=fewer times; 0=never)
6. Do you spray your house with insecticides? (Y/N)

   If yes, what type of insecticide do you use? (Open text box)
7. Do you keep any animals inside your house? (Y/N)

   If yes, which? (Response options: 1=cattle; 2=pigs; 3=dogs; 4=cats; 5=chicken; 6=goats; 7=sheep; 88=other (Open text box))
8. How do you treat jiggers in your family? (Response options: 0=I don't know; 1=extraction with sharp instruments; 2=application of agrochemical substances; 3=application of BBE; 4=application of dimeticone; 88=other (Open text box))
9. If you treat by extraction,
   - do you boil the extraction instrument? (Y/N)
   - do you apply an antiseptic before extraction? (Y/N)
   - do you share the instrument with other people? (Y/N)
   - do you apply anything to the wound? (Y/N)
     - if yes, what do you apply? (Open text box)
10. Do you treat your animals against jiggers? (Y/N)

    If yes, what do you use to treat animal jiggers? (Response options: 0=I don't know; 1=extraction with sharp instruments; 2=application of agrochemical substances; 3=application of BBE; 4=application of veterinary pesticides; 88=other (Open text box))
